# Challenges when Combining Expertise to Provide Integrated Care for Youth At-Risk and Their Family: A Qualitative Study

**DOI:** 10.1007/s10488-024-01430-x

**Published:** 2025-01-23

**Authors:** Laura C.M. Veerman, Eva A. Mulder, Robert R.J.M. Vermeiren, Lieke van Domburgh, Anne van der Maas, Laura A. Nooteboom

**Affiliations:** 1 LUMC Curium - Department of Child and Adolescent Psychiatry, Leiden University Medical Center, Post Box 15, Leiden, 2300 AA the Netherlands; 2 Academic Collaborative Youth At-Risk, Post Box 53, Nijmegen, 6500 AB The Netherlands; 3 Child and Adolescent Psychiatry & Psychosocial Care, Amsterdam University Medical Centre, Meibergdreef 5, Amsterdam, 1105 AZ The Netherlands; 4 Child development and Education, University of Amsterdam, Post Box 15780, Amsterdam, 1001 NG The Netherlands; 5 iHUB, Rotterdam, Netherlands; 6 Lokalis, Utrecht, Netherlands

**Keywords:** Expertise, Youth at-risk, Families, Mental health, Qualitative study, Integrated care

## Abstract

The needs of youth at-risk and their families, facing multiple problems and serious mental health issues, exceed the expertise and possibilities of a single stakeholder (professional, organization, municipality). These youngsters require care in which the expertise of different professionals and organizations is integrated. However, combining various types of expertise to provide integrated care to youth at-risk is challenging. Therefore, this qualitative study aims to describe how stakeholders approach these challenges when combining different types of expertise to provide integrated care for youth at-risk. In total, 28 in-depth interviews were conducted with stakeholders working in various organizations or municipalities for youth at-risk in The Netherlands. Transcripts were analyzed through reflexive thematic analysis. Reflections from a youth representative were also incorporated. Results reveal challenges at four levels: youth and family, professional, organization, and system. At each level, challenges arise in addressing the exceptional needs and problems of youth at-risk, collaborating with multiple stakeholders, reluctance to apply or involve expertise, and finding sufficient resources to combine expertise. Professionals, organizations, and municipalities approach these challenges with a backward or forward approach: either they accept the situation, focus on their own expertise, leave responsibility to others; or they force their expertise on others, or seek collaboration to combine expertise. Overall, combining different types of expertise to provide integrated care to youth at-risk can be seen as an expertise in itself, and necessitates reflection, awareness, and careful consideration from all stakeholders.

**Supplementary Information:**

The online version contains supplementary material available at 10.1007/s10488-024-01430-x.

## Introduction

Youth at-risk face multiple complex problems across various domains, including school dropout, lack of stability and safety at home, and serious mental health issues that pose them at risk for themselves or their environment (Assan et al., 2008; Broersen et al., 2020). These problems often occur simultaneously and mutually influence each other (Hawkins, 2009). Accordingly, their problems exceed the expertise of a single professional, service, or organization (Van den Steene et al., 2019). To address the broad range of needs, a holistic focus is required, encompassing both a generalist view on the entire family’s welfare and a specialist focus on the individual needs of the youngster (Nooteboom et al., 2021). Consequently, combining different types of expertise is indispensable for providing holistic and comprehensive care to youth at-risk (Van den Steene et al., 2019). Earlier studies show that combining different types of expertise enables professionals to enhance their capabilities, address situations collectively, and lead to a comprehensive view of the problems of youth at-risk (Dagenais et al., 2008; D’Angelo et al., 2020; Lim & Wong, 2018; Ziviani et al., 2013). However, combining different types of expertise proves to be challenging, and insight into approaches to face these challenges is lacking.

Expertise is defined as the combination of knowledge and skills someone has in a specific domain (Bradley et al., 2006; Lethbridge, 2019). Accordingly, professionals in youth care possess different types of expertise; for example, while one specializes in safety issues within a family, others have expertise in mental health problems in children or adults. To apply one’s expertise, this is as much a social as an individual process, shaped by, for example, what we have learned or are mandated to do, but also by our interactions with clients, their families, and other members of the care network (Hood, 2012). Hence, expertise is developed through knowledge gained from training and education, as well as through the experience of working with others (Hood et al., 2017). Specifically, when providing integrated care to youth at-risk, expertise is demanded not only within individual professional boundaries, but also in collaborative practice (Hood et al., 2016).

Four interrelated levels are crucial when it comes to combining expertise to provide integrated care for youth at-risk and their families: youth and family, professional, organizational, and system. First, the level of youth and family is described as organizing family-centered care within a unified procedure that spans various domains and fields of expertise (Valentijn et al., 2013). Challenges at this level arise as stakeholders are reluctant to involve additional expertise, for example, due to the difficulties youth and their families have in trusting services or the varying nature of their problems (Broersen et al., 2020; Hood, 2014; Young et al., 2020).

Second, the professional level refers to collaborations between professionals with various expertise to deliver comprehensive care to a specific population (Valentijn et al., 2013). Challenges identified on this level include, for instance, being unaware of the limits of one’s own expertise (Okamato, 2001), not involving expertise timely and adequately (Nooteboom et al., 2021), or difficulties in interprofessional collaboration (Fitzpatrick et al., 2023; Timonen-Kallio, 2019). There is also a risk of losing specific expertise, as interprofessional collaboration can dilute a professional’s focus on their own unique expertise (Van den Steene et al., 2019).

Third, combining expertise requires efficient inter-agency relationships (organizational level), and fourth, a policy focused on coherent care (system level). The organizational level is characterized by challenges such as distrust in expertise, lack of specific skills, conflicting interests, and unclear expectations (Lim & Wong, 2018; Timonen-Kallio, 2019). On the system level, studies address difficulties in coordinating and establishing a cohesive youth care landscape (Blanken, 2024; Phaswana & Erlank, 2023).

Overall, combining different types of expertise on the levels of youth and their families, professional, organization and the system is crucial, yet challenging. Achieving integrated care for youth at-risk necessitates understanding of and awareness on how challenges across these four levels are handled in practice. Previous research showed that there are challenges at multiple levels, but no prior studies have explored these challenges across all levels. Additionally, to our knowledge, we lack insight on how these challenges are addressed in practice. Therefore, this qualitative study aims to describe how professionals, organizations, and municipalities approach the challenges that occur in practice when combining different types of expertise to provide integrated care for youth at-risk. This qualitative study identifies challenges across multiple levels and describes the approaches taken to address these challenges.

## Method

### Study Design

This qualitative study is part of a participatory action research project “From paper to practice” of the Academic Collaborative Youth At-Risk on providing integrated care to youth at-risk, that started in September 2020. The Academic Collaborative Youth At-Risk is a partnership of care services, universities, colleges, experts by experience and municipalities, with the aim to conduct practice-based research. In this project, professionals, policymakers, and a youth representative participated as advisors in every step to ensure co-creation: setting goals, and concretize methods, discussing preliminary findings, and directing further research steps. Furthermore, we organized different reflexive learning sessions to discuss the preliminary findings with the participants of the research and relevant stakeholders. The input from co-creation and these sessions contributed to the direction of the research.

The Medical Ethics Review Board of Leiden University Medical Centre judged that the overall research project should not be subject to evaluation based on the Medical Research Involving Human Subject Act (WMO) and complied with the Netherlands Code of Conduct for Research Integrity. In the overall research project, we conducted interviews, observations, and learning sessions, upon which choices for this specific study were based. For this specific study, we conducted semi-structured interviews and analyzed the data using reflexive thematic analysis (see 2.6). The reporting of the study methods and results was informed by the Consolidated Criteria for Reporting Qualitative Research (COREQ).

### Setting

In The Netherlands, municipalities are responsible for acquiring and organizing the youth care landscape, therefore ensuring the availability of expertise. Within each youth care region, different organizations operate, including community-based support teams, specialized and residential care organizations, and child protection services. Our study focused on four of the 42 youth care regions. In these four regions, in April 2020, an expert team was established as part of a broader partnership involving three residential youth care institutions. Eight professionals with diverse expertise on youth at-risk, including clinical psychology, coaching, and family therapy, aim to enhance accessibility and integrating expertise from residential youth care and minimize out-of-home placements or transfers. This initiative aligns with the broader trend of promoting the integration of various types of expertise in youth care teams (Janssens et al., 2010; Nooteboom et al., 2020). Although there are slight differences in how the youth care landscape is organized in each region, overall, the expertise available and working approaches are comparable. Therefore, and in line with the purpose of this study, we chose to look at challenges and approaches across regions.

### Participants

Purposive sampling was used to select a diverse range of participants for the interviews (Palinkas et al., 2015). We used a multi-level approach to ensure a comprehensive understanding of the different levels (Valentijn et al., 2013). We selected the stakeholders for our sample based on the criterium that participants had worked as a professional, coordinator (i.e. organizational representative), or policymaker for youth at-risk in one of the four youth care regions described in the setting (see 2.2). Participants eligible for participation were invited to participate in semi-structured interviews by one of the researchers (LV) via email. Participants were informed of the study’s objectives and interview procedures in the invitation, and subsequently provided written informed consent before the interview took place. Interviews were conducted solely between the interviewer(s) and respondent, and depending on the preference of the respondent, were scheduled either online via Microsoft Teams (*n* = 12) or at the respondent’s workplace (*n* = 16).

### Data Collection

Qualitative semi-structured interviews were taken for detailed description of the experiences of respondents in their own words, as combining expertise is a dynamic process which cannot be summarized on a single scale (Palinkas et al., 2011). Semi-structured interviews were conducted between May and October 2022 by a trained interviewer (LV), with assistance from another researcher (VW) in two of the interviews. The interviews were guided by a topic list with open-ended questions to facilitate deep understanding of viewpoints and experiences (Palinkas et al., 2015). The topic list was formulated in advance, based on previous research (i.e. Janssens et al., 2010; Nooteboom et al., 2020; Nooteboom et al., 2021; Timonen-Kallio, 2019) and primary findings from the overall research project (see 2.1). The topic list was supplemented with input from reflexive meetings with the research team (LN, EM), two professionals working in the setting, and a youth representative (AM). Subsequently, the topic list was pilot tested on a participant who met the inclusion criteria. The topics included, for example, defining expertise, and differences in expertise (see Appendix A for the topic list). All interviews were conducted in Dutch, audio-recorded, and transcribed verbatim to avoid interpretation bias. The duration of the interviews varied between fifty minutes and two hours (m = 80.5 min). No participant expressed interest in commenting on the transcripts. A learning session with the interview participants and their colleagues (*n* = 22) was organized in February 2023, to reflect on the preliminary results of the interviews. This session also served as a member check. The quotes presented have been converted from Dutch to English by two researchers (LV, CB).

### Authors Reflectivity

The professional background of the researchers was diverse, with a background in psychiatry, criminology, law, and psychology. Two authors (RV, LvD) have experience in serving on the boards of youth care institutions. As part of the research project, the first author (LV) became as action researcher familiar with the way of working within the setting. Hence, there was some degree of acquaintance between participants and the interviewer (LV).

### Analysis

All transcripts were analyzed using reflexive thematic analysis, as it allows to narrate on how personal experiences are located within wider contexts (Braun & Clarke, 2006, 2021). The process consisted of six phases (Byrne, 2022). First, the researcher (LV) got familiar with the data by re-reading and manually coding all transcripts. Second, codes were attached in two rounds to quotes that could be meaningful considering the research questions with an inductive approach. To enrich the interpretation, the transcripts of four interviews were coded by two researchers (LV, JA). Third, the coded data was reviewed and analyzed through reflection meetings with the research team (LV, LN, EM, RV, AM) for potential themes. Fourth, the candidate themes and coded data were reviewed (LV), under supervision of an experienced qualitative researcher (LN). This was an iterative process that involved returning to the interviews to verify if the emerging themes worked in connection with the data. Fifth, the themes were defined through a deep analysis of the underlying codes. During this phase, challenges and approaches were formulated, based on reflections on respondents’ own behavior, or how they have seen others respond in certain situations. As this was often overlapping, we did not distinguish this in our analysis. In the sixth and final phase, themes were examined to ensure they were connected in a logical and meaningful manner by the research team (LN, EM, RV, LvD). Simultaneously, a reflection of a youth representative (AM) was incorporated in Appendix B to deepen the presented narrative.

## Results

### Demographics

Out of 43 stakeholders invited for the interviews, five refused mostly due to time constraints, one was sick at the time of the interview, and nine did not respond. In total, 28 respondents participated in this research. See Table 1 for an overview of their demographics. Most participants were female (*n* = 25), this ratio reflects the actual gender representation of professionals working in youth care in the Netherlands.

**Table 1:** Demographics of participants

Variable	
**Gender**	
Male [*n* (%)] Female [*n* (%)]	3 (10.7%) 25 (89.3%)
**Age**	
Mean age in years [SD] Age range in years	45.8 (11.14) 27–63
**Years of work experience current organization**	
Mean in years [SD] Range in years	3.8 (4.49) 0.33–22
**Organization**	
Municipality [n (%)] Community-based support teams [n (%)] Child protection services [n (%)] Regional table [n (%)] Specialistic youth care providers [n (%)] Expert team [n (%)] Residential youth care organizations [n (%)]	5 (17.9%) 4 (14.3%) 4 (14.3%) 2 (7.1%) 2 (7.1%) 8 (28.6%) 3 (10.7%)
**Function**	
Community-based support team worker [n (%)] Behavioral scientist community-based support team [n (%)] Therapist [n (%)] Behavioral scientist child protection service [n (%)] Coordinator treatment residential care [n (%)] Consultant Expert team [n (%)] Coordinator or manager [n (%)] Policymaker [n (%)]	1 (3.6%) 3 (10.7%) 2 (7.1%) 4 (14.3%) 3 (10.7%) 6 (21.4%) 4 (14.3%) 5 (17.9%)
**Division per region**	
Region 1 Region 2 Region 3 Region 4 Supra-regional	3 (10.7%) 4 (14.3%) 3 (10.7%) 6 (21.4%) 12 (42.9%)

### Findings

**Fig. 1:**
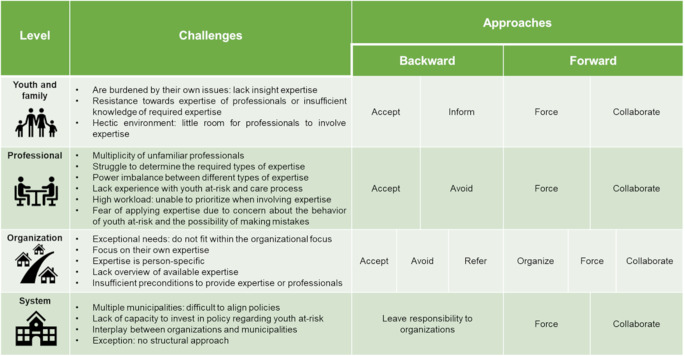
Overview of results

The findings from our reflexive thematic analysis are divided into the four levels described in the introduction: youth and family, professional, organization and system. Figure 1 presents an overview of the results. At each level, we first describe which challenges occur in practice when combining expertise, and then illustrate how these challenges are being approached in practice. Reflections from a youth representative on the results are outlined in Appendix B.

Overall, in dealing with these challenges, stakeholders adopt two overarching approaches, which emerged from our inductive analysis: a backward and a forward approach, each outlining different actions. In the backward approach, stakeholders distance themselves from the challenging situation, for example, by avoiding or accepting it. In the forward approach, stakeholders proactively seek to overcome the challenge, for instance, through forcing or collaborating. Moreover, combining expertise was mentioned as essential at each level. However, at the youth and family, professional, and organization levels, there are limits to the degree to which combining expertise is desirable. Hence, as expertise is person-specific, participants described the importance of critically examining which expertise is needed in the care of youth at-risk, considering the burden on families and the involvement of multiple professionals and organizations.

#### Youth and Family Level: Challenges

Our analysis reported three reasons why combining expertise is challenging on this level. First, most frequently described (*n* = 14), youth and their families cannot (properly) determine what expertise is needed due to the burden of their own issues or care and/or lack of understanding of their own issues. This can result in discrepancies between the perceived needs of youth at-risk and their families, and professionals during the care process, leading to indecisiveness, resistance, stagnation, and uncertainty regarding the required expertise. Second, several respondents (*n* = 8) mentioned youth at-risk and their families do not consent to share information between professionals or (unconsciously) withhold relevant information because they show resistance (i.e. do not take professionals’ expertise seriously) or have insufficient knowledge about the care involved. Finally, four respondents mentioned that the hectic environment around (the care for) youth at-risk and their families, including crisis situations, led to professionals often experiencing little room to involve expertise to avoid overburdening the youth and/or family.

#### Youth and Family Level: Approaches

In the **backward approach**, two actions were identified. First, respondents mentioned that professionals ***accept*** the family’s decision if they decline permission to contact other professionals or lower their expectations and endure there is little room to combine expertise (*n* = 5). Second, professionals ***inform*** the young person and family about how they combine expertise (*n* = 13). For this, it is crucial for the family to acknowledge the expertise of professionals. The **forward approach** also comprised two actions. First, over half of the respondents (*n* = 16) described that expertise is ***forced*** upon the family; professionals see themselves as the experts and determine how and what expertise is needed. Forcing is seen as a last resort to get the development of these youngsters and their family moving in the right direction. In this approach, families are often hardly involved as their involvement could lead to an additional burden for youth and their family. However, respondents often mentioned a lack of clarity about the youngster’s actual wishes and the tendency of youth at-risk and their families to avoid care. Second, respondents described dealing with challenges at the youth and family level by combining expertise in active ***collaboration*** with the young person(s) and family (*n* = 25): the youth and family are the experts on their own lives. Professionals actively and consistently approach the young person and family as an integral part of the care process. It requires a tailored approach to encourage youth at-risk and their family to be open to actively collaborate, such as presenting them with choices or let them make a plan based on the available expertise.

“Each professional interprets it from his or her own perspective, there is nothing wrong with that. But that does, I think, color the perspective, and you really want the perspective of the parents.” - Respondent Expert team 6.

When professionals collaborate with youth and their families to overcome challenges in combining expertise, time and tranquility are important enablers. Moreover, professionals need to be responsive to resistance of families, and have certain skills, knowledge, support, and determination regarding (the behavior of) youth at-risk and their family. To motivate families in collaboration, their (in)formal relationships are helpful. Furthermore, it is crucial to explain why certain expertise needs to be involved and what this means for the care process. This is specifically important when introducing child protection services or residential care providers.

#### Professional Level: Challenges

Our analysis led to six reasons why combining expertise is challenging on the professional level. First, almost all respondents (*n* = 24) described that many professionals involved in the care for youth at-risk are unfamiliar with each other, leading to difficulties in involving required expertise, including hesitance to involve expertise, negative views on each other’s expertise, and unclear decision-making processes. Personal issues between professionals, such as different opinions, norms and values, also challenge combining expertise, as it is time consuming and difficult to call each other to account.

“I really feel that sometimes working with other professionals requires more time, than parents and the client.” – Respondent specialistic youth care provider.

Second, professionals struggle in deciding what expertise is needed because of the multitude, interrelatedness, and unpredictability of problems of youth at-risk and their families possess, resulting in stress, uncertainty, frustration, and difficult alignment between professionals (*n* = 15). Third, respondents (*n* = 18) illustrated that there is a power imbalance or hierarchy between professionals, complicating collaboration between different types of expertise and leading to distrust.

“Why working with [child protection service] is so hard to get going? Well, because there is a kind of an expertise battle and hierarchy. Who is in charge? Who should point out responsibilities to whom? That’s underneath it all.” - Respondent Expert Team 2.

Fourth, it appeared that multiple professionals lack experience in working with these youths and their care processes, resulting in difficulties in transcending domains and unawareness of the limits of their own expertise (*n* = 12). Fifth, due to the high workload, professionals lack time to combine expertise, resulting in a lack of priority regarding youth at-risk cases, or a professionals’ expertise erodes as crisis situations get priority (*n* = 10). Finally, respondents (*n* = 14) indicated that professionals fear applying their own expertise when working with youth at-risk, as they are concerned about the aggressive behavior of youth, potential disciplinary complaints, and/or negative media attention surrounding cases involving youth at-risk. To prevent this, professionals tend to focus on risk averse and controlling measures.

“There is a fear of making mistakes. I mean, she could possibly die (…). This fear limits everyone to just do what they have to do.” - Respondent Regional Table 1.

#### Professional Level: Approaches

On the professional level, the **backward approach** comprehends two actions. First, five respondents described that professionals sometimes ***accept*** that they lack time to combine expertise, and there is a possibility that things go wrong in youth at-risk cases. It helps to accept if you have knowledge of each other’s expertise, are open about your doubts, and address when facing difficulties. Second, respondents (*n* = 17) reported that professionals ***avoid*** each other, focus on their own expertise, and leave when it exceeds their expertise, thus avoiding situations where they must combine expertise. As a result, professionals do not see their own blind spots, and the perspective of the youngster(s) and their family gets lost. Within the **forward approach**, there are also two actions. First, respondents (*n* = 4) described that professionals try to ***force*** their own expertise to be the leading view in a case, with conflicts between professionals as a result.

“Yes then I start overruling, that’s right. Because I think: ‘No, but that is really not helpful for the child’.” - Respondent residential care 2.

Second, almost all respondents described that professionals actively seek ***collaboration*** with each other to overcome challenges in combining expertise (*n* = 26). Collaboration can enhance confidence in their own expertise, prevents a tunnel vision and can help less experienced professionals to work with this challenging group. Collaboration requires effort and investment from professionals, including fixed and regular meetings (i.e. supervision or working in buddies). Other enablers are using connecting language, understanding each other’s expertise, and valuing each other’s expertise as equal. In addition, respondents mentioned that combining expertise in active collaboration with other professionals can be seen as an expertise itself. In that, it is important that professionals have an overview of the entire process, involve and activate the right professionals, and translate various expertise into one comprehensive plan.

#### Organizational Level: Challenges

On the organizational level, five reasons why it is challenging to combine expertise are outlined. First, the care needs of youth at-risk and their families exceed the expertise of a single organization: their needs vary and they often form an exception within the current care system (*n* = 15). Accordingly, these needs make it challenging to determine what expertise is needed, and to find a particular set of services. Second, organizations do not automatically seek collaboration with each other, as they often primarily focus on their own expertise, and interests. Moreover, sometimes organizations are funded under different laws and regulations (*n* = 21). Specifically with these youth, respondents described that pressure from media and governments to comply to rules increases an inward focus based on control and rules. This leads to blind spots within organizations, limited access, false expectations, and resistance towards expertise of other organizations. Third, expertise was described as person-specific: it matters who you call within an organization what expertise is available as you trust the expertise of a person not of an organization (*n* = 22). Besides, respondents mentioned that individuals within and across organizations are often unfamiliar with each other and staff turnover impacts the available expertise.

“We have so many providers. Let’s say we have 200 providers, and all those providers employ 50 people. So looking for expertise is a pure gamble.” - Respondent Regional Table 1.

Fourth, organizations lack insight into available expertise due to the multitude of organizations, which often operate across multiple municipalities, each with its own youth care landscape (*n* = 22). Respondents described it as a “jungle”; they are drowning in the amount of expertise available and it is complicated for organizations to determine the boundaries of their own expertise. Finally, respondents (*n* = 12) described a lack of preconditions to provide expertise for youth at-risk, such as insufficient capacity and staffing problems, leading to uncertainty about available expertise.

#### Organizational Level: Approaches

At this level, the **backward approach** encompasses three actions. First, respondents (*n* = 15) described that organizations ***accept*** they do not know or are unfamiliar with each other’s expertise as it is not feasible to be aware of all available expertise. Moreover, respondents saw it as a fact that not all expertise is available for this exceptional group, and organizations have different interests and make different assessments of expertise. Transparency helps to ensure realistic expectations of other organizations.

“And yes well the thing is, I can’t determine for the mental health department that they don’t do the admission, the psychiatrist has decided to do the admission. Yes, then the choice is made. I must accept it (…). Sometimes you can’t prevent that certain decisions are made by other parties.” - Respondent community-based support team 1.

Second, organizations ***avoid*** each other (*n* = 16): they lack desire to approach other organizations, persist in their own path and are not focused on joint efforts, out of need for overview and efficiency. Organizations hope challenges will disappear naturally, while other organizations are sidelined. Consequently, organizations work alongside each other, and ultimately do not utilize each other’s expertise. Last, organizations ***refer*** cases to other organizations: they decide that their own organization cannot provide expertise, and refer to others, in other words “*the hot potato is passed on*” (*n* = 17). In this approach, organizations lack flexibility and do not experience joint responsibility. To prevent negative perceptions, it helps if organizations properly explain why they refer.

Three actions were identified within the **forward approach**. The first is to ***organize*** a clear work process with different organizations, such as a registration desk or a central point where organizations are integrated (*n* = 24). According to some of these respondents, organizing a work process offers structure to transcend domains, clarity regarding available expertise, a clear and accessible point of contact, and promotes familiarity with each other’s expertise, resulting in short lines of communication between organizations. However, there are also respondents who described that organizing these processes are perceived as “*a must-do*” with no added value and only delays as a result. Professionals feel their own expertise is denied as their input is often not part of the new structure.

Second, respondents described that organizations try to ***force*** their own expertise on other organizations (*n* = 17), occurring in five ways: (1) manipulating (‘If you do not take in this child, your business operations will fail’); (2) assert urgency, (3) blame each other and call out who is responsible, (4) escalating a case to the board of organizations or municipalities, and (5) bypassing established ways of working through shortcuts for placement. While forcing does often lead to care provision, the risk of harm in relationships between professionals is substantial according to several respondents. Moreover, placements are unprepared or rushed, and youth do not feel welcome in an organization. It helps to explain why forcing was used, to personally know each other, and to show mutual respect and understanding.

“We now care for the girl that everyone else in the Netherlands refuses because she shows many externalizing behaviors. We can say: ‘yes, but our team is not quite ready for her and whatever’. However, we are the party that has to step in. So, sometimes you have to put some pressure on them, and we’ve done that now, and that’s not nice, you don’t make friends with that.” – Respondent Expert Team 8.

Third, organizations actively seek ***collaboration*** with each other to overcome challenges when combining expertise (*n* = 25). Not only does this ensure integrated care, but through collaboration, organizations also gain awareness of their own blind spots.

“You don’t know everything, so you really need your colleagues, other expertise and other organizations to be able to give the right help to these clients, because all of them are clients with multiple problems. (…) It goes across all domains.” – Respondent residential care 2.

According to respondents, collaboration is a matter of trial and error, there is no standard procedure. However, respondents mentioned the following enablers: provide a fixed contact person, organize personal and regular contact, work outreaching and proactively, ensure transparency, and demonstrate willingness to invest to a common goal. Another enabler is an open collaborative mindset: dare to look outside existing structures. An important precondition to be flexible as an organization that was described, is financial space and mandate to act. Like the professional level, collaboration between organizations to combine expertise requires additional expertise, demanding independence, decision-making-power, knowledge of different domains and available expertise, ensuring clear roles and tasks, maintaining an overview, and network abilities to ensure each organization feels seen and heard.

#### System Level: Challenges

By law, municipalities are in the Netherlands obligated to ensure expertise is available. Our analysis showed four reasons why municipalities are challenged to organize expertise for youth at-risk. First, supra-regional collaboration is necessary for the exceptional needs of this target group (*n* = 9). Consequently, municipalities need to give up some autonomy and align their local policy to supra-regional plans. Additionally, municipalities have different views and policies on how to organize the local care landscape around expertise, leading to numerous layers between municipalities and practice, resulting in delays and a lack of clarity.

“We have had it quite a few times that someone from the municipality raises his finger saying: ‘This is not good, or that’s not good, and we have to stop it’. Then we have to go all the way back to the beginning: ‘This is a very small target group, it is not doable to organize this type of care at an individual municipality level.’ We also have a common interest in keeping this type of care on a regional level. So that means ‘Dear municipality, we must find common ground and act together’.” – Respondent municipality S3.

Second, municipalities differ in their capacity to put effort in examining the availability of expertise for youth at-risk (*n* = 3). Some policy makers are responsible for the entire youth care landscape, leaving them with insufficient time to invest in the expertise for certain subgroups, such as youth at-risk. Third, there is an interplay between municipalities and organizations (*n* = 5). It is difficult for municipalities to determine what expertise is needed in practice; they need input from organizations. However, one professional described not daring to be transparent with municipalities out of fear of the consequences it might have for her own organization. In addition, municipalities do not always have control over organizations, as organizations can refuse to participate in procurements or prioritize other issues. Fourth, two respondents revealed that there is no structural approach within some municipalities to make expertise available for youth at-risk. It concerns a small number of young people and is seen as the exception, which complicates organizing expertise at the system level.

“I think it’s not sufficient to say [name of organization] gets the whole task, that’s not possible, because there are also other children in the family. So, you need a kind of tandem, a kind of dance of understanding who is the lead (…). But that’s very complicated. In [municipalities] there is one child per year, then you must invent that dance for one child.” - Respondent municipality W2.

#### System Level: Approaches

At this level, the **backward approach** involves municipalities ***leaving responsibility to organizations*** to organize combined expertise (*n* = 7). Municipalities only come into action in case of a complaint or appeal from families. Municipalities provide the budget and stimulate dialogue between organizations. This approach demands collaboration between organizations and requires municipalities to look beyond the boundaries of their system to give financial options to organizations. Organizations vary in their experiences of this approach: while municipalities offer trust in their course, all responsibility is placed on them.

With the **forward approach**, there are two ways in which challenges are approached. First, respondents described that municipalities ***force*** (*n* = 7): the municipality pays, so determines. In this approach, municipalities keep an overview, formulate their own responses to signals from practice, assign responsibilities and procedures, and play an active role in connecting organizations. Organizations must ask permission from the municipality to combine expertise, or regulations are enforced to control costs. Simultaneously, a mandate from government that force municipalities to collaborate is mentioned as an enabler for collaboration between municipalities.

“We as municipalities are not always very keen on that, because I see that they don’t have to go back to the community team at all (..). I think that’s really difficult for [name of organization] that sometimes municipalities… I as a policymaker then have to say: ‘Yes, I understand that it requires more work for your occupation and comes with organizational issues, but I really have a different interest here’.” – Respondent municipality W3.

Second, municipalities actively ***collaborate*** among themselves and with organizations in the field (*n* = 8). The youth care landscape is jointly designed and monitored from a shared vision. Enablers to facilitate collaboration included: commitment, support from the city council(s), regular meetings with organizations and policymakers from various municipalities, and a common goal.

## Discussion

The aim of this qualitative study was to describe how professionals, organizations, and municipalities approach challenges that occur in practice when combining different types of expertise to provide integrated care for youth at-risk. Challenges occur at four levels: youth and family, professional, organization, and system (see Fig. 1). In line with earlier studies (e.g. Phaswana & Erlank, 2023; McLean, 2012), these challenges span from the needs of youth and families, to broader issues within and between organizations and municipalities. For example, challenges emerge when dealing with the exceptional needs and issues of youth at-risk, collaborating with numerous stakeholders, reluctance to apply or engage expertise, a lack of overview of required and available expertise, and establishing adequate resources to combine expertise. Based on our analysis, these challenges are approached in a backward or forward manner. On one hand, professionals, organizations and municipalities accept the situation, follow their own expertise or leave the responsibility to others. On the other hand, they proactively force their own expertise on others, or seek collaboration to combine their expertise. By identifying these approaches, this study can help reflect on and raise awareness of how challenges are addressed, as well as initiate discussions on the impact of different approaches on all stakeholders involved. Based on our results, we have identified two key findings that require further deliberation. First, combining different types of expertise to provide integrated care to youth at-risk can be seen as an expertise in itself, and necessitates awareness in practice. Second, combining expertise demands reflection on which approach enables providing integrated care to youth at-risk in every unique situation.

### An Expertise in Itself

Our results underline that stakeholders struggle to combine the required expertise that is tailored to the needs of youth at-risk. The combination of their needs are exceptional, stakeholders are reluctant to apply expertise out of fear, or lack experience on working with and the care process around youth at-risk. As our study further shows, stakeholders need to combine expertise under pressure and in high-risk and crisis situations, which challenges collaboration with all stakeholders involved. Moreover, various respondents described a need for an additional expertise to support stakeholders in combining different types of expertise in the care for youth at-risk. Therefore, combining expertise to provide integrated care for youth at-risk can be seen as an expertise in itself. Practical implications on what this expertise comprehends is outlined based on the KASH model. The KASH model is known for its impact on the behavior of professionals and organizations as it not only focusses on the Knowledge and Skills, but also by Attitudes and Habits (Vasudevan et al., 2024).

**Knowledge** about the expertise available for youth at-risk, both within and across organizations, is essential to find the necessary expertise for each case. Combining expertise also requires knowledge on the different domains and processes relevant for youth at-risk cases in each region (Blanken, 2024). Especially, because networks that are based on intangible, knowledge-based resources (i.e., information or expertise) tend to be more diffuse than networks based on tangible resources (i.e., contracts) (Huang & Provan, 2007). Simultaneously, an open and willing **attitude** towards the involvement of other types of expertise and equally valuing each other’s expertise is essential, especially when dealing with high-risk and crisis situations, as it enables collaboration and easier alignment (Dagenais et al., 2008). Furthermore, combining expertise for youth at-risk requires **skills** to ensure that the right expertise is combined in practice. Our results show that experience is vital for gaining competence in combining expertise under unpredictable circumstances. The required skills are divided into three areas: (1) obtaining an overview and critically assessing the required expertise by analyzing the needs of youth and their families, (2) translating each type of expertise into a wider context and a single plan, and (3) building relationships with stakeholders to keep all parties involved (e.g., Hood, 2014; Ziviani et al., 2013). Finally, combining expertise requires **habits** to monitor the care process and expertise involved, by regular meetings with relevant stakeholders (Suarez et al., 2012).

A recent systematic review emphasizes the importance for healthcare professionals and organizations in general to bridge gaps between different types of expertise, align overlaps in roles and tasks, and create spaces to facilitate these efforts (Schot et al., 2020). For instance, Interprofessional Education prepares students to think as a team member, and ultimately, understand and appreciate the contributions of each profession to clinical outcomes (World Health Organization, 2010). Although beforementioned shows that combining expertise is a fundamental skill already educated in some areas, the unique combination of problems faced by youth at-risk spans multiple domains and often involves high-risk, crisis situations for which no standard education exists. Future research is recommended to study how, and to what extent, (all) youth care professionals should be trained in combining expertise for youth at-risk, or whether there should be specialists in this particular area.

### Reflection on how Challenges are Approached

Our study shows a variety of approaches in dealing with challenges when combining expertise. Nevertheless, some approaches enable combining expertise to provide care for youth at-risk in practice more than others. For instance, in some cases professionals, organizations and municipalities avoid or force responsibility to others and primary focus on their own expertise. These approaches often appeared where care was not properly delivered to youth at-risk, often with devastating effects (Hood, 2015). Moreover, professionals and organizations can lack insight into their blind spots, potentially leading to inequality in referring and providing care (IJsselhof et al., 2024). This calls for reflection in practice: why are certain approaches used, and what is the impact at each level. A tailored approach from all stakeholders to uncertain and sometimes unpredictable conditions is needed as youth at-risk fall between specific responsibilities of different agencies (Bevington et al., 2013; Hood et al., 2016). A clear implication for practice, based on our results, is the importance of acknowledging the expertise of others and being explicit, transparent, and challenge the considerations behind choosing for a particular approach in a given situation. This necessitates alignment between stakeholders. To realize this alignment, our results have demonstrated the significance of familiarity among relevant stakeholders. However, it also raises questions regarding feasibility in practice. As stated in this study and previous work of Blanken et al. (2023), it is not realistic to know all relevant stakeholders, given the multiplicity and limited resources to invest in these relationships. Hence, this requires explicit awareness from governments and municipalities on how the youth care landscape is organized, as it often consists of many fragmented parties (see Wu et al., 2015 for a practical framework).

### Strengths and Limitations

The qualitative and descriptive nature of this study allowed to unravel the complexity of combining expertise for youth at-risk in practice. Whereas earlier studies focused on the challenges that occur, this study contributes to existing knowledge by describing how challenges are approached, ultimately aspiring to overcome them. Furthermore, our analysis has revealed a consistent and deepened narrative, experienced by a diverse range of respondents across various organizations, regions, and functions, thereby enhancing credibility (Watkins, 2012). Our research is further comprehensively and transparently described following the COREQ checklist, improving transferability (Tong et al., 2007). An additional strength is the engagement of a youth representative and other stakeholders throughout all stages of this study, ensuring relevance for current practice.

Nevertheless, there are limitations. Youth and their families were not included as respondents in this study as this was beyond the scope of our research project. Further research is required to gain insight in how these youth and their families approach these challenges themselves, and how they experience the approaches found in this study. Furthermore, the reported approaches were experiences mentioned by respondents and not based on direct observations of practice, in which (unconscious) behavior can be noted. Besides, this study encompasses a wide variety of respondents who differ in their working experiences within a specific organization and region. As some organizations were just starting, such as the expert team, this could have led to different perceptions and experiences.

## Conclusion

This qualitative study demonstrates challenges that occur when combining expertise to provide integrated care to youth at-risk, and how these are approached in practice. These challenges span from the needs of youth and families to the broader issues within organizations and municipalities. It emphasizes the parallels in the backward and forward approaches between professionals, organizations and municipalities when facing challenges to combine expertise for youth at-risk. Awareness and reflection on these approaches is crucial to combine expertise and thereby providing integrated care. Overall, combining expertise to provide integrated care for youth at-risk can be seen as an expertise in itself, and demands careful consideration from all stakeholders involved.

## Electronic Supplementary Material

Below is the link to the electronic supplementary material.

Appendix A. Topic list interviews

Appendix B. Reflection of a youth representative
