# Supplementary material for: Challenges when Combining Expertise to Provide Integrated Care for Youth At-Risk and Their Family: A Qualitative Study
Source: Adm Policy Ment Health. 2025 Jan 23;52(3):520–32. doi: 10.1007/s10488-024-01430-x (PMC12133975; doi:10.1007/s10488-024-01430-x)
Supplement: Supplementary file 1 — Appendix A. Topic list interviews [file 10488_2024_1430_MOESM1_ESM.docx]

**Appendix A. Topic list interviews**

A1. Topic list professionals and coordinators

| **Topics** | **Questions** |
| --- | --- |
| Background information | - Age, years working in this function and organization |
| Additional expertise youth at-risk | - Thinking freely about your work and in situations where you work with youth at-risk, where would you like additional input or expertise on? |
| Defining expertise | - How would you define expertise? - How do you know what expertise is needed for youth at-risk cases? |
| Using expertise | - When do you think combining expertise adds value? - In what ways do you most easily use someone’s expertise? |
| Considerations expertise | - Suppose you have a case of youth at-risk in which many professionals are already involved at the time, how do you decide whether or not to involve expertise in this case? - What considerations do you make about whether or not to add expertise? - In what ways do you combine different types of expertise in to one? |
| Difference expertise | - How does the expertise of [organizations] relate to your own? - And of your own organization? - And those of professionals already present in the family? - How do you safeguard your own expertise and the of other professionals already involved in the family? - When the advice/suggestions/ideas, for example of [name organization] differ from those of professionals who have been involved with the family for a long time, how do you ensure that all the expertise is combined into a whole in such a case? |
| Approaching situations combine expertise | - How do you know at what point you should call in the expertise of another organization? - How much space do you have to combine expertise at the moment when there is crisis, high tension or pressure in a youth at-risk case? - Who decides whether or not to engage expertise from another organization? |
| Youth and their family | - What’s the family’s role in whether or not to combine expertise? - To what extent does the family play a role in deciding whether or not to engage expertise from another organization? |
| Trust expertise | - To what extent do you trust your own expertise? - To what extent do you have trust in the expertise of other organizations? Why is that? |
| Reflection interview | - Finally, would you like to add anything? - How do you feel about the interview? |

A2. Topic list policymakers

| **Topics** | **Questions** |
| --- | --- |
| Background information | - Age, years working in this function and organization |
| Role municipality in relation to youth at-risk and their families | - How do you see the role of the municipality in providing youth care to youth at-risk, given that, especially in small municipalities, at most 1 or 2 youth at-risk per year live in those municipalities? |
| Role municipality integrated care | - To what extent does the municipality play a role in connecting various organizations? - What role do you think the municipality should ideally play in term of achieving good cooperation between organizations and achieving integrated care? |
| Defining and organizing expertise | - How would you define expertise? - When has someone in youth care expertise? - How does the expertise of the municipality relate to the expertise of professionals in practice? - If you look at how it is organized in your municipality, who do you think has the expertise to determine what is needed in the family and thus whether care can and should be provided? - What is the role of municipalities in that? |
| Approach towards practice | - To what extent do you invest in realizing good collaboration between organizations in practice? - When do you leave it to organizations (again) and under what conditions? |
| Trust expertise | - To what extend do you have trust in the (expertise of) organizations you have to collaborate with? |
| Finance | - To what extent does expertise play a role for municipalities in determining whether or not to provide financial resources for youth care? - How do you deal with the choices organizations make for certain care and the financial impact this has on a municipality? - When you disagree with a referral made from an organization, how do you deal with that? Considering that the care is too expensive and from your perspective not required. |
| Collaboration with practice | - What do you do as a municipality the moment a case is scaled up? So that contact is made with the municipality at the moment that organizations cannot work it out on their own. - How do you handle it when there is a disagreement between municipality and practice? What helps or hinders in search for a solution therein? |
| Reflection interview | - Finally, would you like to add anything? - How do you feel about the interview? |
