# Supplementary material for: Challenges when Combining Expertise to Provide Integrated Care for Youth At-Risk and Their Family: A Qualitative Study
Source: Adm Policy Ment Health. 2025 Jan 23;52(3):520–32. doi: 10.1007/s10488-024-01430-x (PMC12133975; doi:10.1007/s10488-024-01430-x)
Supplement: Supplementary file 2 — Appendix B. Reflection of a youth representative [file 10488_2024_1430_MOESM2_ESM.docx]

**Appendix B. Reflection of a youth representative**


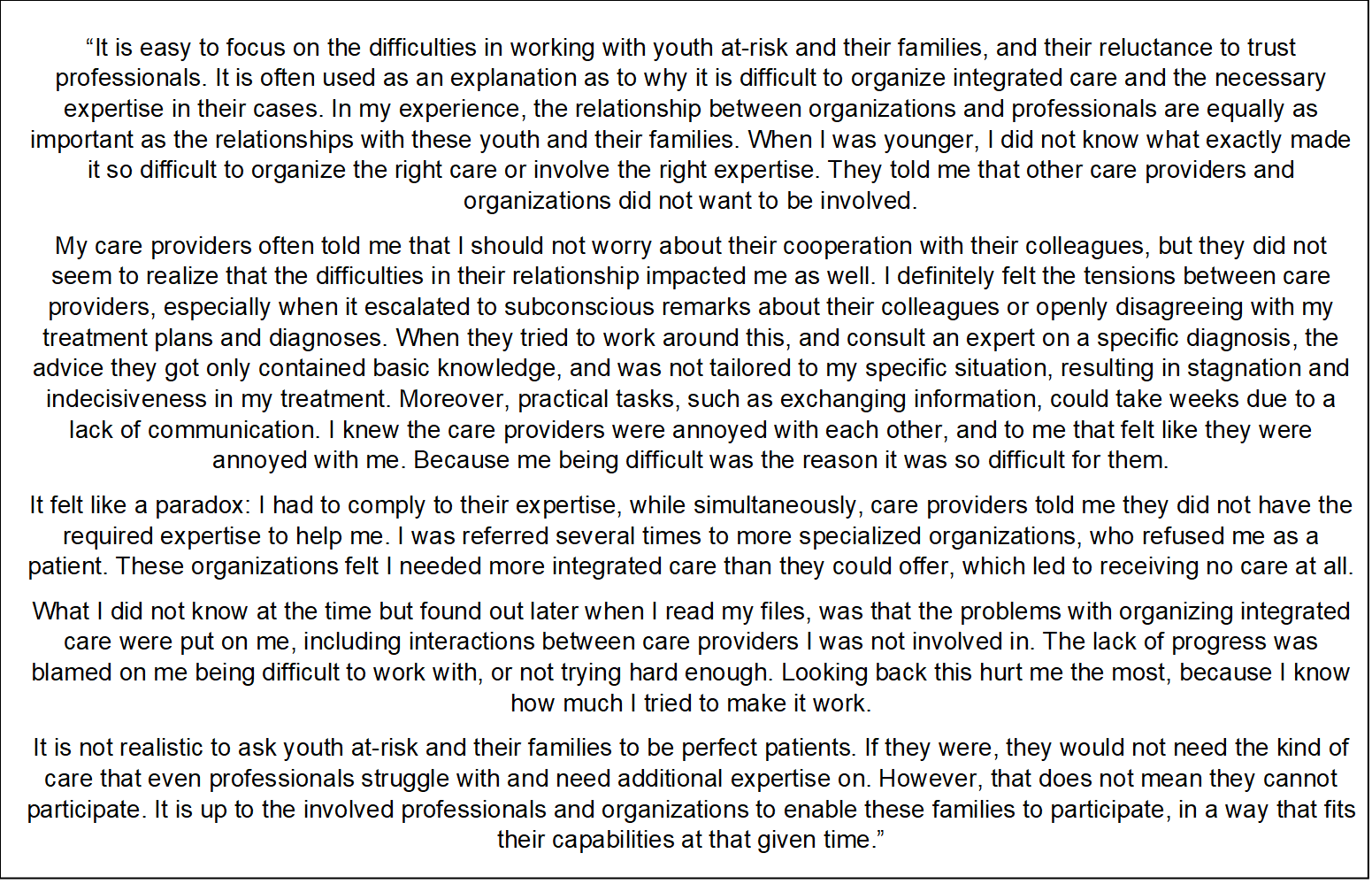
The results presented are based on the experiences of professionals, coordinators and policy makers. In Figure 2, a youth representative reflects on these results.

**Fig. 2** Reflections from a youth representative
